# Preparation of Nano Silver Paste and Applications in Transparent Electrodes via Electric-Field Driven Micro-Scale 3D Printing

**DOI:** 10.3390/nano10010107

**Published:** 2020-01-05

**Authors:** Hongke Li, Xiaoyang Zhu, Zhenghao Li, Jianjun Yang, Hongbo Lan

**Affiliations:** Shandong Engineering Research Center for Additive Manufacturing, Qingdao University of Technology, Qingdao 266520, China; LHK1164072308@163.com (H.L.); 15634218355@163.com (Z.L.); yjjdem@163.com (J.Y.)

**Keywords:** nano-silver paste, micro-scale 3D printing, silver mesh, transparent heaters

## Abstract

Nano-silver paste, as an important basic material for manufacturing thick film components, ultra-fine circuits, and transparent conductive films, has been widely used in various fields of electronics. Here, aiming at the shortcomings of the existing nano-silver paste in printing technology and the problem that the existing printing technology cannot achieve the printing of high viscosity, high solid content nano-silver paste, a nano-silver paste suitable for electric-field-driven (EFD) micro-scale 3D printing is developed. The result shows that there is no oxidation and settlement agglomeration of nano-silver paste with a storage time of over six months, which indicates that it has good dispersibility. We focus on the printing process parameters, sintering process, and electrical conductivity of nano-silver paste. The properties of the nano-silver paste were analyzed and the feasibility and practicability of the prepared nano-silver paste in EFD micro-scale 3D printing technology were verified. The experiment results indicate that the printed silver mesh which can act as transparent electrodes shows high conductivity (1.48 Ω/sq) and excellent transmittance (82.88%). The practical viability of the prepared nano-silver paste is successfully demonstrated with a deicing test. Additionally, the experimental results show that the prepared silver mesh has excellent heating properties, which can be used as transparent heaters.

## 1. Introduction

Electronic paste is an electronic functional material that integrates metallurgy, the chemical industry, and electronic technology. With the rapid development of electronic information technology, the demand for flexible and stretchable electronics, electromagnetic shielding, radio frequency identification systems, electronic packaging and interconnections, solar cells, and so forth is increasing rapidly. As a key functional material of such electronic components, electronic paste has attracted extensive attention in its development and application fields [1,2,3,4,5]. At present, the most commonly used materials for the preparation of electronic pastes are conductive polymers [6,7], metal nanoparticles [8,9,10], carbon nanotubes, and graphene [11,12,13,14]. The overall performance, especially the conductive properties of carbon-based materials and conductive polymers materials, is much lower than that of metallic materials. Among the metal-based materials, gold has the best conductivity, but the price of gold is relatively high and the reserve content is low, which limits the further application of gold powder. The price of copper and aluminum is relatively low and both of them have excellent electrical conductivity, but copper powder and aluminum powder are easily oxidized during storage, and the electrical conductivity is also reduced, which limits their applications in electronic paste. Compared with other conductive materials, silver powder not only has excellent electrical conductivity but also a good chemical stability and relatively lower price. Thus, silver powder is the preferred material for preparing various electronic pastes [15,16,17,18].

To date, a series of strategies have been developed for the fabrication of silver powder, such as the mechanical ball milling method, the light induced method, the template method, the liquid phase reduction method, and so forth [19,20,21]. For instance, Wang et al. reported that monodisperse silver nanoparticle conductive ink was successfully synthesized by an in-situ synthesis method in an aqueous solution [22]. The results showed that the silver ink can be successfully printed on cotton fabric and the highest conductivity was 2 × 10^−5^ Ω·m when the silver content was 30 wt%. Jiu and coworkers [23] fabricated a micron-sized silver paste through using a new solvent composed of 4-(tert-butyl) cyclohexyl acetate as a dilute agent and a new material as a thickener through chemical reaction. The electrical resistivity of silver patterns with 3 × 10^−4^ Ω·m was obtained by sintering at 280 °C for 30 min. Fang [24] prepared the silver paste by using silver powders as the conductive phase, m-phenylenediamine, terpineol, and epoxy resin as the adhesive, and polyethylene glycol as a surfactant. The paste can be used in screen printing and cured at 150–180 °C for 30 min to make a strong combination with the substrate. The resistance of the fabricated silver conductive line can be as low as 4 × 10^−4^ Ω·m. Lei [25] developed an electrohydrodynamic (EHD) jet printing strategy based on in situ reactive inks to fabricate microscale conductive silver features. The printed features mainly contain nanoscale silver particles and exhibit an electrical conductivity of 3.3 × 10^−4^ Ω·m. At present, nearly all the nano-silver pastes and nano-silver inks are developed according to the matching of printing and manufacturing processes. Whether it is contact printing technology (screen printing, intaglio printmaking) or non-contact printing technology (aerosol jet printing, inkjet printing), different printing technologies have different performance requirements for different printing materials. Especially for some applications that require high performance, silver pastes with high solid content and low sheet resistance are required. For contact printing technology, such as screen printing technology, although silver paste with high viscosity can be patterned, screen printing cannot achieve the printing of high-resolution silver wire. At the same time, the process of screen printing is tedious, the mask needs to be replaced once for each printed circuit, the cost is relatively high, and there is a problem of material waste [26,27]. For non-contact printing technology, such as inkjet printing technology, it is only suitable for the printing of silver inks with low viscosity (generally less than 30 mPa·s) and cannot achieve the printing of silver pastes with high viscosity and high solid content [28,29]. Although EHD jet printing can achieve the printing of high viscosity materials, this technology has requirements for the conductivity of the printing substrate, it has certain limitations for non-conductive printing substrates. Thus, it is difficult to achieve a high-resolution printing of high solid content silver paste on non-conductive and thick substrates [30,31,32]. Additionally, the serious agglomeration phenomenon for the silver paste with high solid content is very common and difficult to solve, which may easily cause the blocking of the nozzle during EHD jet printing and affects the stability and continuity of printing.

Aiming at the shortcomings of the existing nano-silver paste and the problems that the existing technology cannot achieve, such as the stable and continuous printing of high viscosity or a high solid content nano-silver paste, we develop a nano-silver paste with a content of 75% by weight, good dispersing properties, and electrical properties. Meanwhile, electric-field-driven (EFD) micro-scale 3D printing technology is used to print the silver paste to verify the printing characteristics of silver paste [33,34]. In this paper, we discuss and analyze the fabrication details and properties of the silver paste and the printing characteristics of silver paste via EFD micro-scale 3D printing technology.

## 2. Experiment Details

### 2.1. Materials

All of the chemical reagents used in the experiments were of analytical grade and used as raw materials without further purification. These were: silver nanoparticles (the size of silver powder was about 300 nm, (Flance (Beijing) Nanotechnology Co., Ltd. (Beijing, China)), epoxy e44 (Sinopharm Chemical Reagent Co., Ltd. (Beijing, China)), alpha-terpineol (Sinopharm Chemical Reagent Co., Ltd. (Beijing, China)), ethanol (Sinopharm Chemical Reagent Co., Ltd. (Beijing, China)), ethyl cellulose (Sinopharm Chemical Reagent Co., Ltd. (Beijing, China)), dibutyl phthalate (Shanghai Macklin Biochemical Co., Ltd. (Shanghai, China)), polyethylene glycol 40000 (Sinopharm Chemical Reagent Co., Ltd. (Beijing, China)), silane coupling agent KH570 (Dow Corning (Shanghai) Co., Ltd. (Shanghai, China)), hydrogenated castor oil (Sinopharm Chemical Reagent Co., Ltd. (Beijing, China)), polyvinyl pyrrolidone (Sinopharm Chemical Reagent Co., Ltd. (Beijing, China)), succinic acid (Shanghai Macklin Biochemical Co., Ltd. (Shanghai, China)).

### 2.2. Preparation of Nano-Silver Paste

The preparation process of nano-silver paste should be carried out in a relatively clean environment to avoid mixing in other impurities during the preparation process, which will affect the performance of the nano-silver paste and cause pollution. In this experiment, the whole process of preparation of nano-silver paste was as follows:

First, was the pretreatment of silver powder: We dissolved the succinic acid to a certain proportion (The weight ratio of succinic acid to ethanol is 1:16.) in the ethanol solution to obtain the succinic acid-ethanol solution I, then the pre-weighed silver nanoparticles are poured into the solution I for ultrasonic waves and stirred treatment for 30 min, and the layers are separated by centrifugation to obtain the treated silver nanoparticles. The ratio of the weight percentage of silver nanoparticles to that of the succinic acid is 1:1. Succinic acid contains a carboxyl group which can act as the dispersant to prevent aggregation of the silver nanoparticles efficiently.

Next, the alpha-terpineol, water-free alcohol, and polyethylene glycol 40,000 were selected as the solvent (the solvent proportions of 6:2:1, respectively), and the ethyl cellulose, dibutyl phthalate, hydrogenated castor oil, polyvinylpyrrolidone, silane coupling agent KH570, were used as the pasting agent, plasticizer, leveling agent, dispersing agent, and coupling agent, respectively. The above reagent was fully mixed in the proportion of 5:5:3:3:2, ethyl cellulose was dissolved in a constant temperature water bath at 50 °C and the whole process was carried out in a stirring and ultrasonic environment to fully dissolve the mixture in the beaker. In addition, since different solvents have different boiling points, they show different volatile properties at different temperatures zones, hence different solvents were selected as organic solvents. The epoxy E44 was slowly added into the mixture for 20 min and then mixed with the mixed solution in the proportion of 10:9 to get the organic vehicle.

Finally, the pretreated silver nanoparticles were slowly added to the mixed organic vehicle in batches and ultrasonically stirred for 120 min. Then, the nano-silver paste was ball milled for 48 h using a ball mill to obtain a nano-silver paste with excellent performance. The mass ratio of silver nanoparticles to a mixed organic vehicle was 7:3.

### 2.3. EFD Micro-Scale 3D Printing Technology

**Figure 1:**
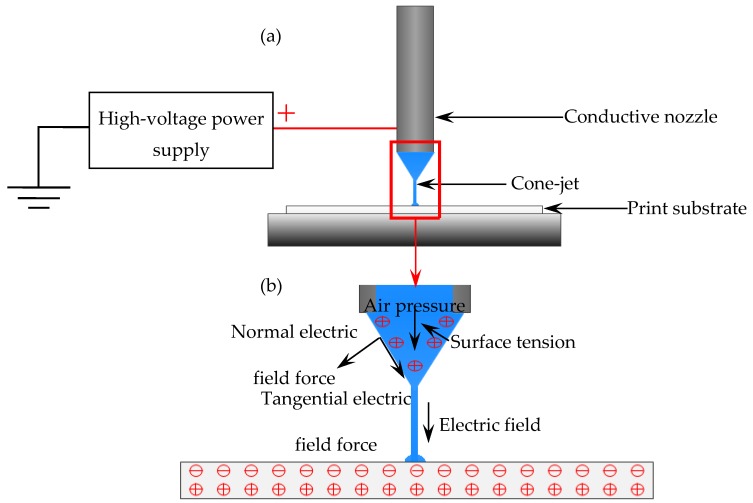
Schematic principle of the electric-field-driven (EFD) micro-scale 3D printing: (**a**) EFD micro-scale 3D printing overall structure; (**b**) the electrostatic induction between the nozzle and substrate and stresses acting on the meniscus.

In this paper, the proposed EFD micro-scale 3D printing technique is an improved printing method based on the electrostatic induction and EHD cone-jetting, which is different from the traditional pressure-driven 3D printing and the existing EHD jet printing [35,36]. As shown in Figure 1a, only the conductive nozzle is required to be connected to the positive pole of the high-voltage power supply, and the grounded substrate is no longer needed as the counter electrode. The electrode interacts with the substrate, causing charge redistribution on the substrate surface when a print head moves close to the target substrate with its nozzle connected to a positive electrode in the power supply. As a result, a stable electric field can be generated between the nozzle and the substrate. During the applied voltage signal, the meniscus is stretched and deformed gradually to form a Taylor cone under the combined action of the electric field force, viscous force, air pressure, and surface tension, as shown in Figure 1b. As the liquid or fused material breaks through the surface of the Taylor cone, a diameter-reducing jet flow is ejected onto the target substrate. The stable electric field forming and the printing process is not limited by the substrate material. It can directly print electronic components or conductive circuits on different substrates (flexible substrates, rigid substrates, and even stretchable substrate).

### 2.4. Characterization and Measurements

The morphologies and structures of the silver nanoparticles and conductive films were characterized by field-emission scanning electron microscopy (FE-SEM, MERLIN Compact Germany). Furthermore, we inspected the elemental composition and distribution of conductive silver films through energy-dispersive X-ray spectroscopy (Energy dispersive spectrometer, EDS, Germany) analysis by using the Electron Backscattered Diffraction, EBSD equipped in the FE-SEM. For the electrical characteristics of the conductive film, we utilized the four-point probe (Four-point probe, ST2258C, Suzhou Jingge Electronic Co., Ltd. China) resistance measurement system to measure sheet resistance. Optical transmission of the silver meshes was determined using a UV-vis spectrophotometer (UV-6100, Shanghai metash instruments Co., Ltd. China) in the wavelength range of 400–800 nm with a step size of 1 nm. The relationship between the quality and temperature of the nano-silver paste was characterized by the thermogravimetric (TG) and differential scanning calorimeter (DSC), (STA449F3 Jupiter, Germany).

## 3. Results and Discussion

### 3.1. Testing Results of the Nano-Silver Paste

#### 3.1.1. Stability of Nano-Silver Paste

**Figure 2:**
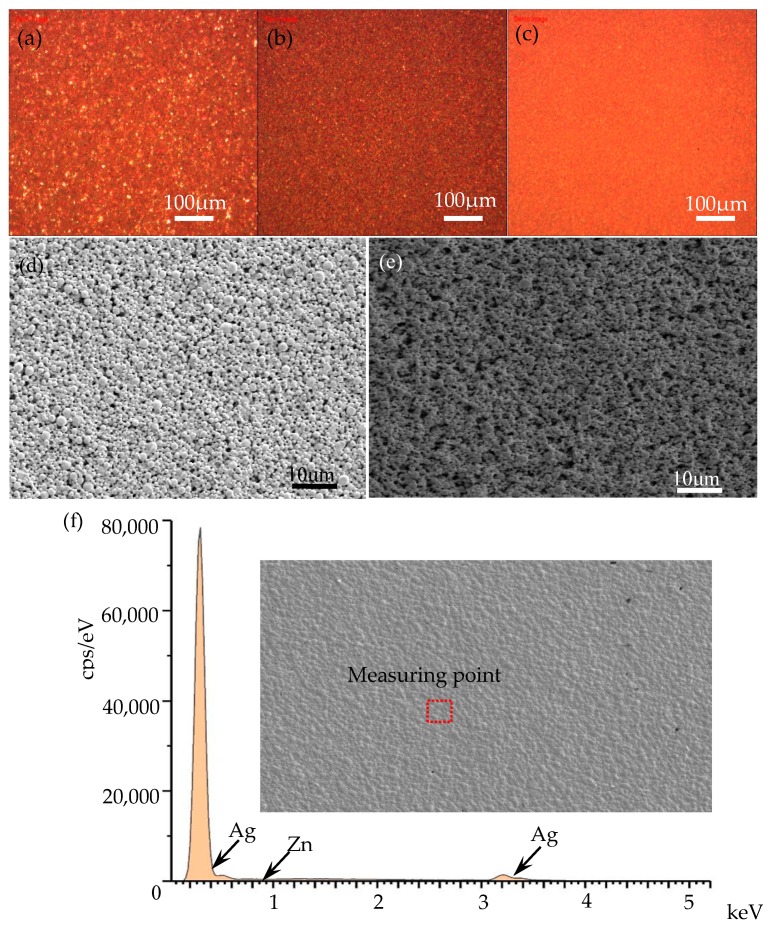
Microstructure and morphology of nano-silver paste: (**a**,**b**) are the optical microphotos of two different silver pastes purchased from the market; (**c**) the optical microphoto of nano-silver paste prepared in this work; (**d**,**e**) are the SEM of nano-silver pastes with a time interval of more than six months; (**f**) energy dispersive spectrometer analysis of nano-silver paste.

The stability of the nano-silver paste is an important indicator to determine its application value. In this study, we compared the other products to observe whether there is sedimentation or agglomeration of nano-silver particles in the nano-silver pastes stored for more than six months, as shown in Figure 2. Figure 2a,b show the nano-silver pastes on the market, Figure 2c shows the nano-silver paste prepared in this work. The stability of the nano-silver paste is an important indicator to determine its application value. According to Figure 2a, we found that there are many large-sized silver particles in the nano-silver paste, the distribution of silver particles is uneven and there is an agglomeration of silver particles. During the printing process, the existence of these unfavorable factors blocks the printing nozzle, which is not conducive to high-resolution printing. The situation of the nano-silver paste in Figure 2b is improved, and the distribution of silver particles is relatively uniform, but there are still a few large-size silver powders. In addition, the surface of the nano-silver paste in Figure 2a,b is relatively rough compared to the micromorphology of the nano-silver paste in Figure 2c under the natural leveling state, the silver particles have a uniform size and good compactness in Figure 2c. Figure 2d,e show the SEM of nano-silver pastes with a time interval of more than six months. According to the results, we found that the silver particles in the nano-silver paste were evenly distributed without the agglomeration of nano-silver paste with a storage time of over six months, which indicates that it has good dispersibility.

Moreover, energy dispersive spectrdmeter (EDS) was utilized to identify and characterize the elemental composition of silver films after storing for a long period. A typical EDS spectrum taken on the silver film is shown in Figure 2f. According to Table 1, we can see that the content of the Ag element is the highest, accounting for 99.78%, and a small amount of Zn element with content of about 0.22% is also doped. These results indicate that the nano-silver paste has a high antioxidant capacity and excellent stability in storage time. This is because such merits of good oxidation resistance and long-term stability for silver films are associated with the polymer protectant utilized during the preparation process of silver nanoparticles. This polymer protectant can protect silver nanoparticles from oxidation in the air, since it attaches to silver nanoparticles and forms a protective coating on the surface of the silver nanoparticles, which can reduce exposure to air efficiently and improve long-term storage stability.

#### 3.1.2. The Sintering and Curing of Nano-Silver Paste

**Figure 3:**
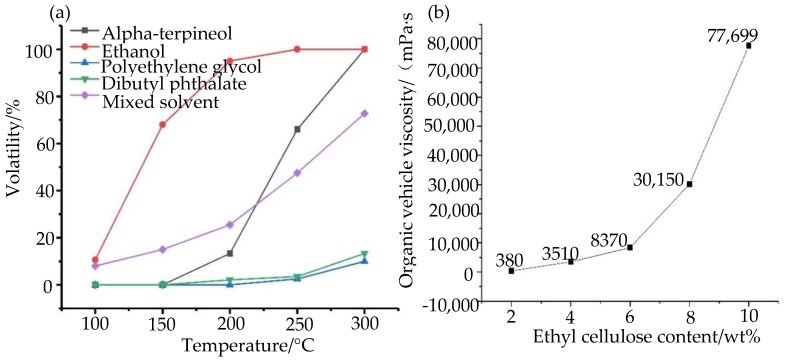
Performance study of the organic vehicle: (**a**) volatility of organic solvent; (**b**) effect of ethyl cellulose content on viscosity of the organic vehicle.

During the preparation of the nano-silver paste, on the one hand, the organic vehicle is required to have different volatilization rates to ensure that the nano-silver paste has a good leveling state in different stages. On the other hand, it is also necessary to ensure the viscosity of the organic vehicle, as suitable viscosity can not only prevent the sedimentation of the nano-silver paste but also adjust the rheological properties of the nano-silver paste to achieve high-resolution printing. For this reason, we studied the performance of organic vehicle, as shown in Figure 3. Figure 3a shows the volatility of different organic solvents at different temperatures, and Figure 3b shows the effect of ethyl cellulose content on the viscosity of the organic vehicle.

In this study, we weighed a certain amount of alpha-terpineol, ethanol, dibutyl phthalate, polyethylene glycol 40,000, and their mixed solutions in a beaker and measured the weight via electronic scales. Then, the temperature of the vacuum drying box was set to 100 °C, 150 °C, 200 °C, 250 °C, and 300 °C. After the above five kinds of organic solvents were placed at different temperatures for 30 min, the remaining weight was weighed. We repeated the experiment more than three times and calculated the average value of the results of the experiment to obtain the amount of solvent volatilization. According to Figure 3a, it can be seen that the volatility of ethanol is the largest. When the temperature reaches above 250 °C, the ethanol has completely evaporated. The volatility of the polyethylene glycol 40,000 is the lowest and the volatility of the dibutyl phthalate is similar to that of the polyethylene glycol 40,000. The volatility of the alpha-terpineol is between three single solvents, and the terpineol is completely volatile when the temperature reaches 300 °C. Since different solvents have different boiling points, different solvents exhibit different volatility in different temperature ranges. In the process of preparation of organic vehicle, if a single solvent is used to prepare organic vehicle, especially the solvent with strong volatility, the prepared organic vehicle will not be stored for a long time. In contrast, if the nonvolatile solvent is used, the electrical properties of the nano-silver paste will be affected by the residue of organic solvent after sintering and curing. According to Figure 3b, with the increase of the proportion of ethyl cellulose, the viscosity of the organic vehicle also increases. The experimental results show that the phenomenon of sedimentation and stratification will occur when the content of ethyl cellulose is less than 4 wt%, which cannot be stored for a long time. When the content of ethyl cellulose is more than 10 wt%, the silver powder is difficult to disperse uniformly because of the high viscosity of the organic vehicle, which easily causes the agglomeration of silver powder. After repeated experiments, it was found that the prepared nano-silver paste had the best performance when the content of ethyl cellulose was 6–8 wt%, and the phenomenon of sedimentation and stratification no longer occurred.

**Figure 4:**
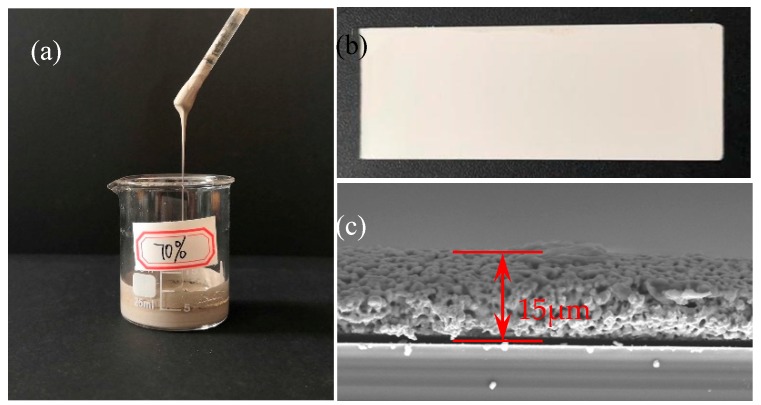
Sample piece for the test of sheet resistance: (**a**) the prepared nano-silver paste; (**b**) test sample; (**c**) the cross section of the sample of SEM.

In order to investigate the influence of sintering and curing process parameters on the sheet resistance of nano-silver paste, the sintering time and its temperature were varied. As shown in Figure 4, we prepared the thin film sample with the ordinary slide glass as the substrate to measure the sheet resistance of the nano-silver paste, in which the silver content of the nano-silver paste was 70 wt%. Figure 4a shows the appearance characteristics of the prepared nano-silver paste, Figure 4b shows a prepared nano-silver film based on a slide glass, Figure 4c shows the SEM picture of the cross section of the sample, and the thickness was measured to be 15 μm.

**Figure 5:**
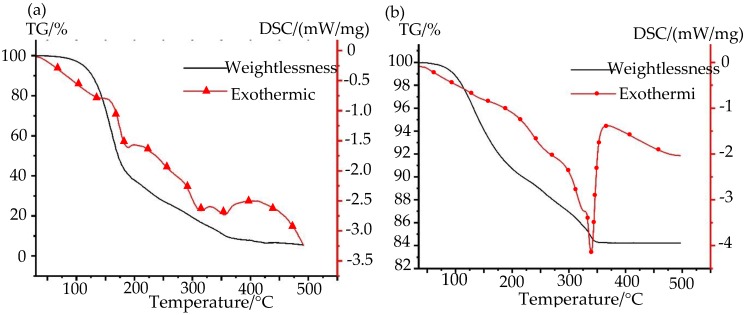
Thermogravimetric analysis (TG) and differential scanning (DSC) calorimetric analysis. (**a**) TG-DSC analysis of the organic vehicles; (**b**) TG-DSC analysis of the nano-silver paste.

The sintering curing process is an important factor to determine the electrical conductivity of the nano-silver paste, which does not have the conductive ability before sintering curing. Therefore, the nano-silver paste can obtain the best conductivity only under the proper sintering curing process. To investigate the appropriate sintering and curing temperature of the prepared nano-silver paste, we carried out a thermogravimetric analysis (TG) and differential scanning calorimeter (DSC) analysis experiment on the organic vehicles and the prepared nano-silver, as shown in Figure 5. It can be seen that the organic vehicle has an obvious weightlessness phenomenon and is accompanied by the appearance of the endothermic peak when the temperature ranges from 100 °C to 400 °C, as shown in Figure 5a. It shows that ethanol and alpha-terpineol in organic solvents are volatilized and decomposed successively, the weight of the sample remains substantially constant when the temperature reaches 400 °C. During the whole heating process, the weight of the organic vehicles was in a relatively gentle state of reduction, which is due to the different boiling points of different solvents in the organic vehicle, therefore the organic vehicle shows different volatilization performances in different temperature ranges. Furthermore, the weight loss rate of the organic vehicle was about 92%, which indicates that some substances in the organic vehicle were not decomposed. This part of the substance is mainly used to connect and support the silver powder in the nano-silver paste to avoid defects such as voids or cracks in the nano-silver paste after sintering. It can also be seen from Figure 5b that a particularly strong endothermic peak appears at a temperature of about 340 °C, which indicates that the organic solvents have a rapid decomposition and volatilization process in this period of temperature. When the temperature is between 50 °C and 350 °C, the weight loss of the nano-silver paste is also gradually reduced in a relatively flat state, and there is no obvious weight loss phenomenon and endothermic peak. This proves that the sintering and curing process of the nano-silver paste is a process of gradual decomposition and volatilization with the increase of temperature, which is conducive to improving the conductivity of the nano-silver paste and to avoiding defects, such as cracks after sintering and solidification of nano-silver paste, due to rapid volatilization at one time.

**Figure 6:**
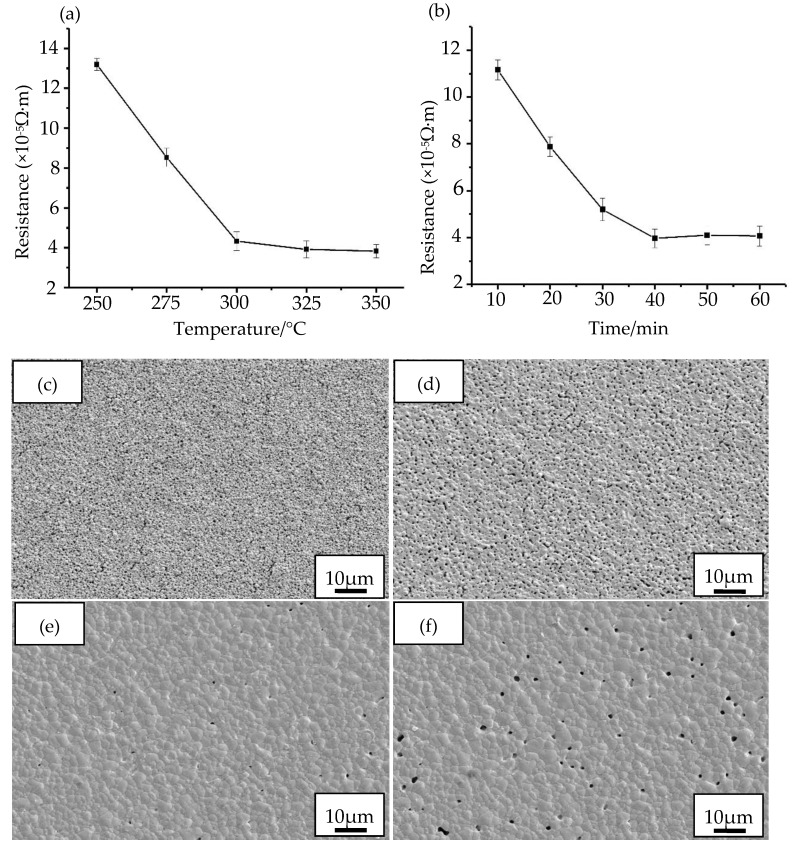
Effect of sintering curing process on the sheet resistance of silver film: (**a**) Temperature: 250 °C, 275 °C, 300 °C, 325 °C, 350 °C; (**b**) Time: 10 min, 20 min, 30 min, 40 min, 50 min, 60 min; (**c**–**f**) the SEM images of silver films with different sintering times (10 min, 20 min, 40 min, 60 min) for 300 °C.

Based on the thermogravimetric (TG) and differential scanning calorimeter (DSC) experiment (TG-DSC), we studied the sintering and curing process of the prepared nano-silver paste. Figure 6 shows the variation of sheet resistance of the prepared nano-silver film at different sintering and curing temperatures and times. Figure 6a shows the change of sheet resistance measured by sintering 30 min at temperatures of 250 °C, 275 °C, 300 °C, 325 °C, and 350 °C. Figure 6b shows the change in sheet resistance measured at different sintering times of 10 min, 20 min, 30 min, 40 min, 50 min, and 60 min under the sintering temperature of 300 °C. Obviously, Figure 6a shows that the sheet resistance of the silver film is gradually decreased from 13.19 × 10^−5^ Ω·m to 3.98 × 10^−5^ Ω·m as the sintering temperature is increased from 250 °C to 350 °C. We can see that the changes of silver film resistance tend to be gentle when the sintering temperature reaches above 300 °C. This is because the sintering and curing reaction process has been entirely completed, the organic solvent is substantially volatilized and the silver powder particles are more closely connected. Therefore, the sheet resistance of the silver film can substantially no longer change when the sintering temperature is further increased. For the sintering time, the sheet resistance of the silver film can be changed by increasing the time. Figure 6b shows that the sheet resistance is reduced from 11.16 × 10^−5^ Ω·m to 3.83 × 10^−5^ Ω·m when the sintering time is increased from 10 to 40 min. When the sintering time is more than 40 min, the sheet resistance of the silver film will change slightly. This is because the sintering time is too long to cause a void in the surface of the silver film and a defect in the increase in porosity, which leads to a deterioration of the electrical conductivity. This slight variation of sheet resistance indicates that the sintering time can be shortened. Therefore, the optimal sintering and curing process conditions are selected to be cured for 40 min at the temperature of 300 °C on the basis of the principle of saving resources and reducing costs. Figure 6c–f show SEM images of the fabricated silver films with different sintering times within a range of 10–60 min for 300 °C. We can see that silver nanoparticles of the calcined films are apparently melted and connected with the surrounding nanoparticles as the sintering time rises gradually from 10 to 60 min, the compactness of the silver film gets better and better. We find from Figure 6c that the silver nanoparticles are connected most closely, and the compactness of the silver film is also optimal when the sintering time is 40 min. This can facilitate and enhance the electron transmission between silver nanoparticles. Therefore, the sintering time has a great influence on the electrical conductivity properties of the silver film. As shown in Figure 6f, the probability of defects, such as voids on the surface of silver film, is increased if the sintering time is increased, which is also the reason for the increase of sheet resistance of silver film.

### 3.2. EFD Micro-Scale 3D Printing Results of the Nano-Silver Paste

#### 3.2.1. Geometric Morphology of the Printed Silver Wires

**Figure 7:**
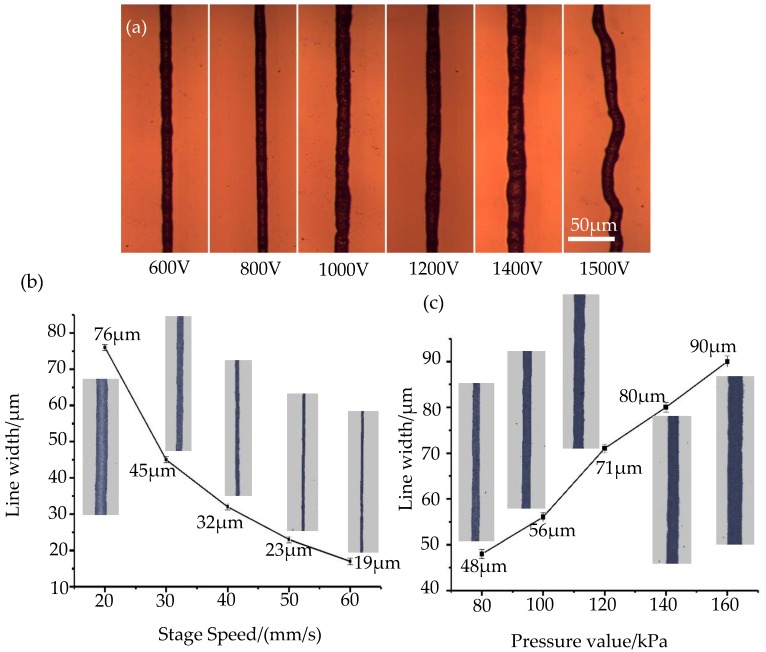
Printing results of the nano-silver paste under different process parameters: (**a**) micrograph of printing results of the nano-silver paste at different voltages; (**b**) the printing results of the nano-silver paste with the printing platform moving speed; (**c**) the printing results of the nano-silver paste with the different air pressures.

In order to verify the feasibility of the prepared nano-silver paste printing, a series of printing experiments is carried out on different substrates (glass, polyethylene terephthalate (PET) and photographic paper) to explore the printing process of the prepared nano-silver paste (Video S1, Supporting Information). During the printing process, the driving voltage is one of the important process parameters that affect EFD micro-scale 3D printing, the driving voltage directly determines the Taylor cone formation and the stability of the printed wire during the printing process. Figure 7a shows the morphological dimensions of printed graphics at different voltages, the driving voltage varied from the smallest value for jetting, the threshold voltage, to the largest value for maintaining normal printing. According to the experimental results shown in Figure 7a, the silver line with good size and morphology can be printed when the driving voltage is increased from 600 to 1400 V. When the driving voltage is above 1500 V, the surface appearance quality and the uniformity of the printed wiring are deteriorated. The main cause of this phenomenon is that when the driving voltage value is too large, the electric field strength at the printing nozzle also increases continuously and a single stable cone-jet state is destroyed, thus leading to a deterioration in the size of the printed pattern. When the driving voltage and air pressure remain unchanged, the line width of the printed wire is determined by the moving speed of the printing platform. When the moving speed of the printing platform is too small, the width of the printed wire is relatively thick, conversely, the amount of liquid discharged cannot keep up with the speed change, which will cause a discontinuous state of the printed wires. In order to investigate the effect of the moving speed of the printing platform on the printing results, the experiment uses glass as the printing substrate, and the diameter of the printing nozzle is 160 μm, the driving voltage is 800 V, the air pressure value is 120 kPa, and the distance from the printing nozzle to the glass substrate is 0.15 mm. Figure 7b shows the changing trend of the moving speed of the printing platform to the forming size. The experimental results show that when the printing speed is increased from 20 to 60 mm/s, the line width of printing decreases from 76 to 19 μm, with the increase of the moving speed of the printing platform, the deposition of nano-silver paste per unit area on the substrate also decreases, hence, the linewidth of the printed wire is also reduced.

The air pressure is the main factor affecting the discharge speed of the printing material. During the printing process, the formation of a continuous and stable jet has certain requirements on the discharge speed, that is, the minimum flow velocity requirement. In order to investigate the effect of air pressure on the printing results, the experiment uses glass as the printing substrate, and the diameter of the printing nozzle is 160 μm, the driving voltage is 800 V, the moving speed of the printing platform is 40 mm/s, and the distance from the printing nozzle to the glass substrate is 0.15 mm. Figure 7c shows the changing trend of the air pressure value and the printed wire forming size. It can be found from the experiments that when the pressure value meets the minimum flow velocity requirements of the printing material, that is, when the pressure value reaches 80 kPa, the printed wire starts to form a continuous line. At this time, the line width of the printed wire is 48 μm. When the printing air pressure value is increased to 160 kPa, the line width of the printed wire is 90 μm. With the continuous increase of the printing air pressure, the line width of the printed wire also increases continually. Therefore, during the printing process, we should select the appropriate air pressure value according to the accuracy requirements of the print size.

**Figure 8:**
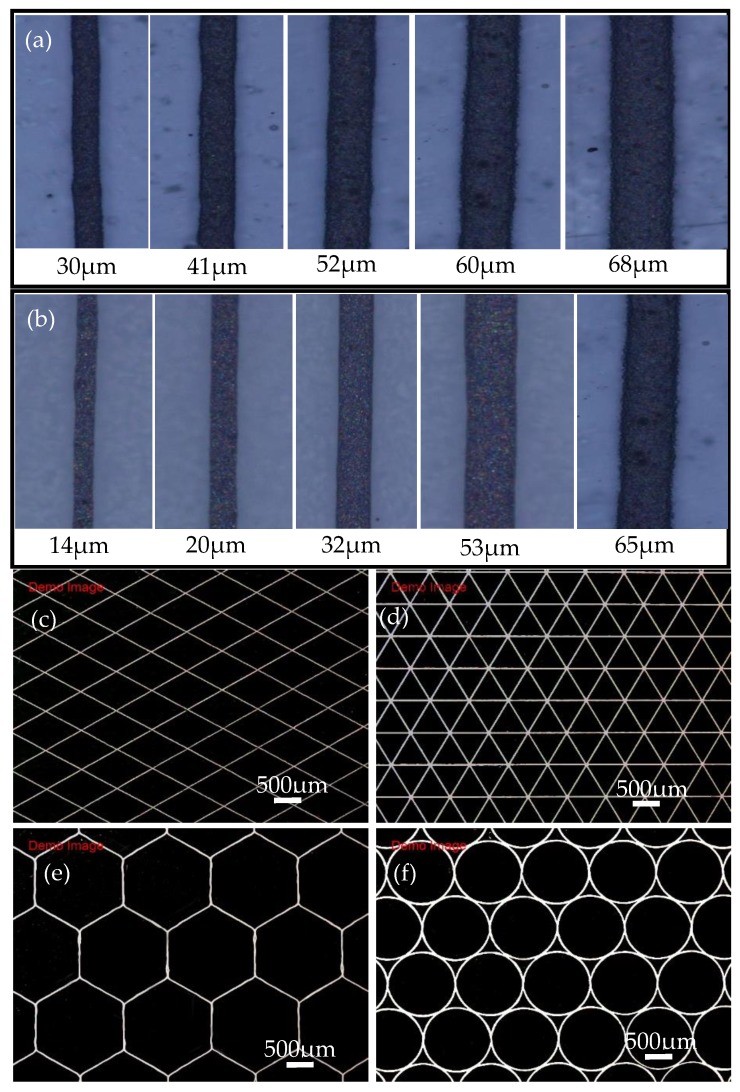
Printing results of nano-silver paste on different substrates. (**a**) Variable line widths printing on the PET substrate; (**b**) variable line widths printing on photographic paper; (**c**–**f**) printed different structure graphics on glass substrate.

In order to further verify the feasibility of printing nano-silver paste on different substrates, the stability of EFD printing of different graphs on flexible PET, photographic paper, and glass is realized, as shown in Figure 8. Figure 8a,b show the printing results of the silver wires on the PET substrate, of which the line width size of printed silver wires ranged from 30 to 68 μm, and the photographic paper, of which the line width size of printed silver wires ranged from 14 to 65 μm. Figure 8c–f show the printed silver wires with rhomboid, triangle, hexagonal, and circular graphics shapes, respectively. The experimental results show that printing silver wires with different widths and structure graphics can be realized by adjusting the different printing process parameters, and the accuracy of the wire forming performance is high and the consistency is good. The application potential of the prepared nano-silver paste in the EFD micro-scale 3D printing technology is verified.

#### 3.2.2. Photoelectric Properties of the Printed Silver Mesh

In order to investigate the photoelectric properties of the printed silver mesh, ordinary float glass was used as printing substrate (size: 100 mm × 100 mm × 2 mm), the printing equipment is an industrial grade EFD micro-scale 3D printer with independent intellectual property rights. The silver mesh with different pitches were printed on glass substrate by using a printing sprinkler with an inner diameter of 160 μm.

**Figure 9:**
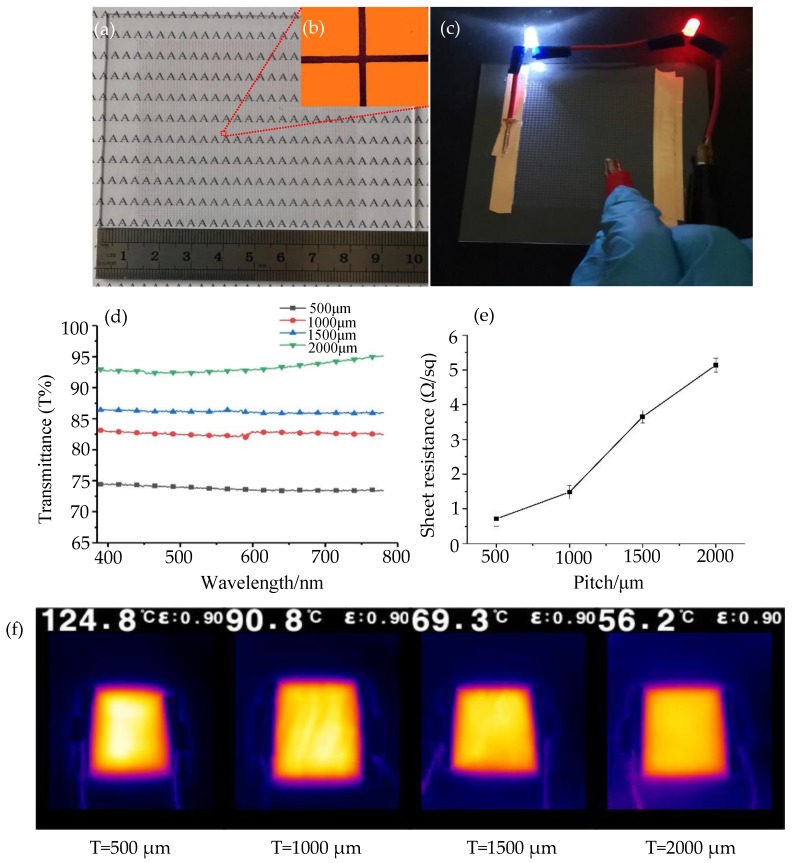
Photoelectric performance test of silver mesh: (**a**) silver mesh electrode structure; (**b**) the micro-morphology of the silver mesh electrode structure; (**c**) conductivity test of silver mesh electrode structure; (**d**) optical transmittance of the silver mesh with different pitch sizes in the visible range; (**e**) variations in Rs with the pitch of the silver mesh; (**f**) the temperature distribution of the silver mesh in different pitches is heated at 5 V for 60 s.

Figure 9a shows the printed silver mesh, (printed area: 70 mm × 70 mm, pitch: 1000 μm, average line width: 30 μm). At the same time, the electrical conductivity of the printed silver mesh was tested, as shown in Figure 9c, and the width and height of the printed lines before and after sintering were measure. The results show that the average shrinkages of the width and height were 1.82% and 3.64%, respectively, which indicates little shrinkage of the printed structure during the sintering (Figures S1 and S2 Supporting Information). The experimental results show that the silver mesh (printed area: 70 mm × 70 mm, pitch: 1500 μm, average line width: 40 μm) electrode structures can light up the LED lamp at different contact positions (Video S2, Supporting Information). Figure 9d shows the spectral transmittance of the silver mesh electrode structures with different pitches, at the incident wavelength of 550 nm, and the transmittance of silver mesh structure electrodes with pitch of 500 μm, 1000 μm, 1500 μm, and 2000 μm are 73.78%, 82.88%, 86.17%, and 92.6%, respectively. The experimental results show that the printed silver mesh (at λ = 550 nm) exhibited good optical transmittance from 400 to 800 nm. Figure 8e presents the mesh Rs as a function of the pitch, measured using a four-probe method and a milli-ohmmeter. Figure 9f shows the heating cloud diagrams of the silver mesh at 5 V for 1 min under different pitches.

According to the test results, with the increase of the silver mesh pitch, the corresponding sheet resistance also increases. The experimental results show that when the pitch of the printing silver mesh is 500 μm, 1000 μm, 1500 μm, and 2000 μm, the corresponding sheet resistance is 0.71 Ω/sq, 1.48 Ω/sq, 3.65 Ω/sq, and 5.14 Ω/sq, respectively. At the same time, we investigated the oxidation and conductivity of the printed silver mesh electrode by measuring the sheet resistance change of the silver mesh (pitch: 1000 μm) before and after the storage time of more than six months, and we measured that the change rate of the sheet resistance of silver mesh with a printing pitch of 1000μm was 3–5%. In addition, the heating performance of the silver mesh electrode structure with a period from 500 μm to 2000 μm was also verified. At 5 V voltage, the maximum temperatures of the silver mesh electrode structure measured by infrared imager are 124.8 °C, 90.8 °C, 69.3 °C, and 56.2 °C after 1 min of power on, respectively, as shown in Figure 9f. The experimental results show that the prepared nano-silver paste has good electrical properties. Therefore, our study for preparing nano-silver paste is undoubtedly very practical and offers the advantages of low cost and high quality for electronic manufacturing.

#### 3.2.3. Practical Application Performance of the Printed Silver Mesh

**Figure 10:**
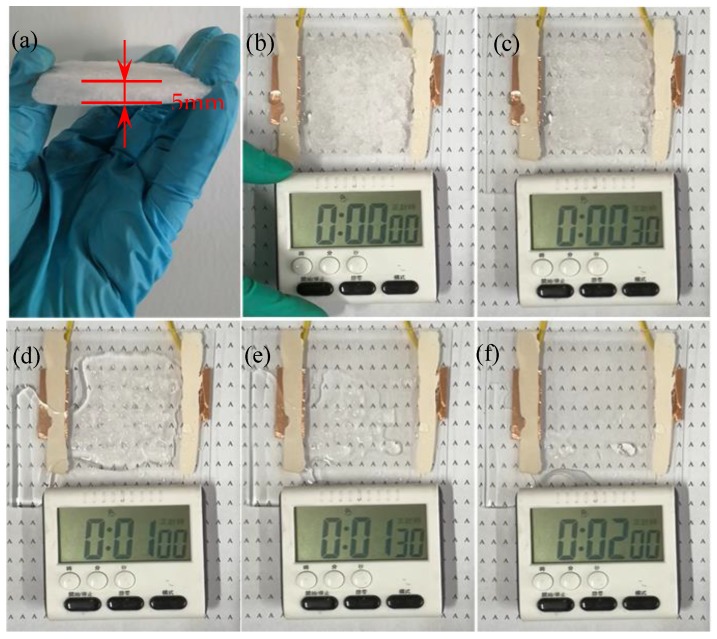
Deicing test: (glass substrate area: 100 × 100 mm, thickness: 2 mm, silver mesh area: 60 × 70 mm); (**a**): a digital image of ice cube with an area of 60 × 70 mm and a thickness of 5 mm; (**b**–**f**): digital photos of deicing at 0, 30, 60, 90, and 120 s.

In order to further investigate the practicability and feasibility of the prepared nano-silver paste, we verified this characteristic performance through a deicing test (Video S3, Supporting Information). Figure 10a,b show that an ice cube with an area of 60 × 70 mm and a thickness of 5 mm was placed on a glass substrate with an electrode structure (silver grid area: 60 × 70 mm, pitch: 1500 μm, width: 30 μm). With the operating at 6 V (DC), the thickness of the ice cube began to melt after 30 s (Figure 10c), after 60 s the ice cube was substantially melted (Figure 10d), after 90 s, the ice cube was completely melted, but a small number of water droplets remained on the glass surface (Figure 10e). The water droplets also evaporated completely after 120s (Figure 10f). The experimental results show that the deicing effect is pretty good at 6V, they prove that the prepared nano-silver paste had good feasibility and practicality.

## 4. Conclusions

In conclusion, we provide a method for preparing nano-silver paste used for EFD micro-scale 3D printing. Additionally, the sheet resistance of the printed film was measured with sintering temperatures. We demonstrated that the electrical conductivity of silver paste can be improved by increasing the sintering time, and it was found that the specific sheet resistance of 3.83 × 10^−5^ Ω·m, which is the lowest value in this work, could be obtained by sintering nanoparticles at 300 °C for 40 min. We realized the printing of different sizes of patterns on different substrates, which verified the feasibility of the prepared nano-silver paste in EFD micro-scale 3D printing technology. Furthermore, the silver mesh electrode structures with different pitches (pitches: 500 μm, 1000 μm, 1500 μm, and 2000 μm) were fabricated by printing the nano-silver paste. The heating test results of the silver mesh (the transmittance and sheet resistance of silver mesh structure electrodes with pitches of 500 μm, 1000 μm, 1500 μm, and 2000 μm are 73.78%, 82.88%, 86.17%, and 92.6% and 0.71 Ω/sq, 1.48 Ω/sq, 3.65 Ω/sq, and 5.14 Ω/sq, respectively) on the glass substrate (thickness = 2 mm) showed that the highest temperature heated by the silver mesh electrode structure was 124.8 °C, 90.8 °C, 69.3 °C, and 56.2 °C after 1 min at 5 voltage, respectively. In addition, the practical viability of the prepared nano-silver paste was demonstrated via a successful deicing test. It proves that the prepared nano-silver pastes have good electrical conductivity and electrical heating performance. Significantly, our prepared nano-silver pastes can be placed at room temperature for more than six months without the oxidation and settlement agglomeration phenomenon. These unique features of the nano-silver paste are particularly useful to many printed electronics applications, such as sensors, photovoltaic cells, and radio frequency identification.

## Figures and Tables

**Table 1:** Main information of EDS energy spectrum analysis of nano-silver paste.

Element	wt%	wt% Sigma	Atomic Percentage/%
Zn	0.22	0.19	0.36
Ag	99.78	0.19	99.64
Total	100		100

## Supplementary Materials

The following are available online at https://www.mdpi.com/2079-4991/10/1/107/s1, Figure S1: Contour changes of silver line before and after sintering, Figure S2: Shrinkage of printed lines before and after sintering Table S1: Main information of EDS energy spectrum analysis of nano-silver paste, Video S1: Electric-field-driven (EFD) microscale 3D printing process of nano silver paste. Video S2: Conductivity testing of printed silver mesh, Video S3: Deicing testing of printed silver mesh.
