# Supplementary material for: Preparation of Nano Silver Paste and Applications in Transparent Electrodes via Electric-Field Driven Micro-Scale 3D Printing
Source: Nanomaterials (Basel). 2020 Jan 5;10(1):107. doi: 10.3390/nano10010107 (PMC7022831; doi:10.3390/nano10010107)
Supplement: Supplementary file 1 [file nanomaterials-10-00107-s001.zip › nanomaterials-682296-supple/supplementary files/supplementary files.doc]

**Supporting Information**

Figure S1. Contour changes of silver line before and after sintering.

Before sintering

After sintering

72 μm

80 μm

140 μm

70 μm

79 μm

138 μm

Shrinkage: 2.78%

Shrinkage: 1.25%

Shrinkage: 1.43%

Figure S2. Shrinkage of printed lines before and after sintering.
